# Prediction Model for Timing of Death in Potential Donors After Circulatory Death (DCD III): Protocol for a Multicenter Prospective Observational Cohort Study

**DOI:** 10.2196/16733

**Published:** 2020-06-23

**Authors:** Angela M M Kotsopoulos, Piet Vos, Nichon E Jansen, Ewald M Bronkhorst, Johannes G van der Hoeven, Wilson F Abdo

**Affiliations:** 1 Department of Intensive Care Radboudumc Nijmegen Netherlands; 2 Department of Intensive Care Elisabeth-TweeSteden Hospital Tilburg Netherlands; 3 Dutch Transplant Foundation Leiden Netherlands; 4 Department of Health Evidence Radboud Institute for Health Sciences Radboudumc Nijmegen Netherlands

**Keywords:** organ donation, tissue and organ procurement, clinical prediction rule, donation after circulatory death, clinical protocols, withdrawal of life-sustaining treatment, end-of-life care, organ transplant, circulatory death, cohort study, intensive care unit, organ donor

## Abstract

**Background:**

Controlled donation after circulatory death (cDCD) is a major source of organs for transplantation. A potential cDCD donor poses considerable challenges in terms of identification of those dying within the predefined time frame of warm ischemia after withdrawal of life-sustaining treatment (WLST) to circulatory arrest. Several attempts have been made to develop models predicting the time between treatment withdrawal and circulatory arrest. This time window determines whether organ donation can occur and influences the quality of the donated organs. However, the selected patients used for these models were not always restricted to potential cDCD donors (eg, patients with cancer or severe infections were also included). This severely limits the generalizability of those data.

**Objective:**

The objectives of this study are the following: (1) to develop a model predicting time to death within 60 minutes in potential cDCD patients; (2) to validate and update previous prediction models on time to death after WLST; (3) to determine timing and patient characteristics that are associated with prognostication and the decision-making process that leads to initiating end-of-life care; (4) to evaluate the impact of timing of family approach on organ donation approval; and (5) to assess the influence of variation in WLST processes on postmortem organ donor potential and actual postmortem organ donors.

**Methods:**

In this multicenter observational prospective cohort study, all patients admitted to the intensive care unit of 3 university hospitals and 3 teaching hospitals who met the criteria of the cDCD protocol as defined by the Dutch Transplant Foundation were included. The target of enrolment was set to 400 patients. Previously developed models will be refitted in our data set. To further update previous prediction models, we will apply least absolute shrinkage and selection operator (LASSO) as a tool for efficient variable selection to develop the multivariable logistic regression model.

**Results:**

This protocol was funded in August 2014 by the Dutch Transplant Foundation. We expect to have the results of this study in July 2020. Patient enrolment was completed in July 2018 and data collection was completed in April 2020.

**Conclusions:**

This study will provide a robust multimodal prediction model, based on clinical and physiological parameters, that can predict time to circulatory arrest in cDCD donors. In addition, it will add valuable insight in the process of WLST in cDCD donors and will fill an important knowledge gap in this essential field of health care.

**Trial Registration:**

ClinicalTrials.gov NCT04123275; https://clinicaltrials.gov/ct2/show/NCT04123275

**International Registered Report Identifier (IRRID):**

DERR1-10.2196/16733

## Introduction

### Background

There is a worldwide shortage of deceased organ donors. Controlled donation after circulatory death (cDCD) is an increasing source of organs for transplantation. In the Netherlands, 59% of the effectuated postmortem organs were from cDCD donors. An increasing number of countries worldwide are establishing a cDCD program. The proportion of organ donations from cDCD donors compared to brain death donors is projected to increase in the upcoming years [1].

However, there are major challenges specific to the cDCD program. First, these patients are not brain-dead and organ donation can only occur after circulatory death (cardiac arrest). As such, withdrawal of life-sustaining treatment (WLST), including stopping mechanical ventilation, should occur to allow circulatory arrest. The time between WLST and circulatory arrest determines whether organs can be donated or not. In most countries, this time is set at a maximum of 1 or 2 hours to preserve organ quality for transplantation purposes. If patients do not arrest within this time frame, organ donation cannot occur. One of the hurdles of cDCD donations is to predict which patients will arrest within the specific time frame. This directly affects family guidance as the treating team and families have to manage family expectations, especially when failure to donate occurs because patients do not arrest within the specified time frame after WLST. Finally, inaccurate prediction of time to circulatory death also influences efficient utilization of the organ procurement and transplantation teams; after WLST is started, these teams need to be fully prepared and present in the operating room to manage recovery and transplantation of organs if the potential donor dies within the given time frame [2].

Factors associated with early circulatory arrest after treatment withdrawal include a younger age, being on artificial ventilation without spontaneous triggering by the patient, needing a high percentage of oxygen, the use of vasopressors, the absence of brain stem reflexes, and a low arterial pH [3]. Interestingly, there are studies that suggest that the use of analgesics or sedatives does not significantly influence the timing of death [4-6].

Several attempts have been made to develop models that predict the time between treatment withdrawal and circulatory arrest [3,7-10]. Two predictive models, the University of Wisconsin Donation After Cardiac Death Evaluation tool and the United Network for Organ Sharing (UNOS) scoring system, were developed in the United States, but neither have been fully validated for practice in European countries [11,12]. The usability of these predictive models is limited as there is a 50% failure of predicting time to death within 1 hour of WLST [13]. The 1-hour time frame is used in many countries as a cutoff to exclude the harvesting of organs from cDCD donors. The DCD-N model, developed from a patient population dying from a neurologic condition, reached 72% accuracy [14-16]. This still means that nearly 30% of patients would not be correctly identified using the DCD-N model. In addition to the limited accuracy, patients with known contraindications to organ donation were included in most previous prediction studies (eg, patients with end-stage cancer and severe infections). This greatly hampers the generalizability of such models [14].

Another important factor that could affect donor potential is end-of-life treatment. The practice of WLST is highly variable between intensive care units (ICUs) and countries [13,17,18]. This influences the dying process and possibly the timing of circulatory arrest. To our knowledge, none of the previously published prediction modelling studies thoroughly assessed the process of WLST. As such, it is unknown if WLST practices could have a major influence on the timing of death in cDCD donors.

Necessary steps to be taken before the initiation of a cDCD procedure are as follows: prognostication, making the decision to withdraw life-sustaining treatment, and approaching family and obtaining their consent for organ donation. Initiation of end-of-life care in acute settings and inexperience in organ donation practices outside ICUs have a negative impact on the number of potential donors [19,20]. Postponing the discontinuation of medical treatment gives professionals more time to guide families adequately and inform them about the dying process and organ donation [21]. Data collected about end-of-life decision-making can provide more insight into whether a patient may be eligible for a cDCD procedure based on time to circulatory death. Such insight can be useful when giving grieving families estimations of time to death and the likelihood that a donation procedure could be performed. However, studies on this important topic are lacking.

### Objectives

The primary objective of this large multicenter study is to develop a model predicting time to circulatory death within 60 minutes in potential cDCD patients. A second important aim is to validate and update previous predicting models on time to death after WLST. Other objectives are to assess the process of end-of-life decision-making, to evaluate the effect of the timing of family approach on consent to organ donation, and to determine the influence of variations of WLST on the timing of death and the corresponding effect on the number of donated organs.

## Methods

### Study Design

This protocol describes a multicenter observational prospective cohort study of all potential cDCD donors of 3 university hospitals and 3 teaching hospitals in the Netherlands. The teaching hospitals were selected based on their diverse focus (including one hospital with cardiologic facilities, one with cardiologic and cardiothoracic facilities, and one with neurosurgical and traumatology facilities), which will result in a highly generalizable cDCD cohort due to the varied patient population admitted to these hospitals.

This study has an observational design and will analyze, without intervention, the characteristics of deceased potential cDCD donors and end-of-life care as provided by the participating hospitals. Therefore, informed consent is not required.

### Participants

The participants of this study are all patients that are admitted at the ICU of one of the hospitals included in this study and are eligible for a cDCD procedure as defined by the Dutch Transplant Foundation [22]. In addition to these organ donation–specific criteria, the following general inclusion and exclusion criteria will be used.

### Inclusion and Exclusion Criteria

Inclusion criteria are the following: (1) patients aged between 18 and 75 years; (2) patients that are mechanically ventilated; and (3) patients in whom medical intervention is not of benefit, resulting in an end-of-life decision. Exclusion criteria are the following: (1) nonintubated patients; (2) patients who are clinically brain-dead but in whom relatives nevertheless specifically requested a cDCD procedure; and (3) patients with contraindications as defined by the Dutch Transplant Foundation, including the following: unknown cause of death, unknown identity, untreated sepsis, malignancy, or active viral infection with herpes zoster, rubella, rabies, HIV, or tuberculosis.

### Data Collection and Management

Data will be prospectively collected by the local investigators, supported by a research manager (International Organization for Standardization certified), and recorded using a web-based electronic case report form (eCRF). The variables will be obtained from the electronic medical records of the following hospitals: Radboudumc Nijmegen, Erasmus University Medical Center Rotterdam, University Medical Center Groningen, Isala Hospital Zwolle, Jeroen Bosch Hospital ’s-Hertogenbosch, and Elisabeth-Tweesteden Hospital Tilburg. Before inclusion, all local investigators received detailed written instructions and on-site training regarding the completion of the eCRF. In addition, the lead investigator team will have regular site visits to perform random sample checks on patient files and data entry. The primary investigator has access to the encrypted data. The primary investigator reviews all incoming data for accuracy and completeness. The research manager can generate data queries and provide trailing records on adjustments of data entered. After completion, the data will be exported in SPSS files (IBM Inc) for further analysis.

A preceding pilot analysis with retrospectively collected data was performed. Two different retrospective data sets were created. One included data from a single hospital (Elisabeth-Tweesteden Hospital, Tilburg, the Netherlands) with a neurosurgical and traumatology focus [23]. The second data set included nationwide demographic data from 2014 to 2016 of all cDCD donors that did not arrest within the set time frame of circulatory arrest of 120 minutes. Anonymized data were provided by the databases of the Dutch Transplant Foundation [24]. Apart from discussion within our own research group, previously published prediction models and analysis of these two data sets contributed to the assessment of key variables to be collected, refinement of the eCRF, defining the appropriate prediction models on time to death for external validation, and providing insight in the donor potential pool after 2, 3, and 4 hours.

Variables to be collected are summarized in Multimedia Appendices 1 and 2. Diagnosis on admission will be classified according to the International Statistical Classification of Diseases and Related Health Problems by the World Health Organization (WHO), tenth revision (ICD-10). Variables related to end-of-life care are shown in Textbox 1.

**Parameters to be collected during and after withdrawal of life-sustaining treatment (WLST).**

- Withdrawal of mechanical ventilation
- Removal of endotracheal or tracheostomy tube
- After endotracheal or tracheostomy tube removal: insertion of an oropharyngeal airway, suction of secretions, lateral decubitus positioning, oxygen administration
- Type and dose of medication administered for palliative care purposes

Variables on neurologic examination; physiological variables; and dose of sedation, analgesia, and vasopressors will be evaluated at 3 time points (1) at time of end-of-life decision-making of the medical team, (2) 30 minutes before WLST, and (3) at 1 time point after WLST until circulatory arrest (Multimedia Appendix 3).

Additionally, the computed tomography (CT) images of the brain of all included patients at admission and the last brain CT prior to WLST will be evaluated using a standardized blinded approach by a neurologist and neuroradiologist. The location and size of brain disorders, magnitude of brain shift, and presence of hydrocephalus will be assessed.

### Statistical Analysis and Sample Size Calculation

First, the patient population will be randomly split in two groups: the first group will consist of 80% of the sample population and will be used for developing the model, while the second group (20% of the sample population) will be used to estimate the performance of the model developed. For the development of a new prediction model, we will apply least absolute shrinkage and selection operator (LASSO) as a tool for efficient variable selection to develop the multivariable logistic regression model. For this, we will use the glmnet library (Version 3.0-2). To find the regulation parameter λ, 20-fold cross-validation is applied. To gain some robustness in the choice for the optimal model, we will use the λ.1se option. As quantification of the model fit, the area under the curve (AUC) from the 20% testing portion of the sample population will be presented.

Previous studies on time to circulatory death in cDCD patients found that approximately 50% to 70% will die within 60 minutes after WLST [3,7,15,23,25]. We estimated that approximately 50% of our cohort will have circulatory death within this time frame, which is balanced to patients that will die after this time frame. The sample size determination for our study was based on including enough patients to reach a sufficient level of precision for the AUC for the 20% testing group. Our goal is to have a 95% confidence interval for the AUC with a width of 0.16. This translates into a standard error of 0.04 for the AUC. With an expected AUC of approximately 0.84 and a population with balanced outcomes (50% mortality within 60 minutes of WLST), this requires a sample of N=100. As we will split our patient population into an 80% development portion and a 20% portion for assessing predictive performance, this study requires the inclusion of 400 patients overall.

Second, previously published prediction models will be externally validated using our data set of 400 patients [7,15,25]. We will use logistic regression models with death within 60 minutes (yes/no) as the outcome. These models will be applied to the validation data and performance of the models will be assessed in terms of discrimination and calibration. The calibration process will consist of three steps. First, a calibration plot will be made for the original predictor. Second, the coefficients of the original predictor will be shrunk by multiplying them by the slope of the calibration curve. For this shrunken predictor, a new calibration curve is fitted. Third, calibration will be completed by adjusting the original intercept with the intercept from the calibration curve with the shrunken coefficients. The models will be refitted in the new data set.

### Ethical Consideration

The Medical Research Ethics Committee Brabant in the Netherlands has approved the study protocol (NW2014-36). The Medical Ethics Committees of all participating hospitals assessed and consented the study protocol. This study is registered at ClinicalTrials.gov with the unique identification number NCT04123275.

## Results

The results of this study are expected to be presented at international scientific meetings and published in 2020 or 2021. The study findings will be reported according to the guidelines outlined by the STROBE (STrengthening the Reporting of OBservational studies in Epidemiology) and TRIPOD (Transparent Reporting of a multivariable prediction model for Individual Prognosis Or Diagnosis) statements [26,27].

## Discussion

An accurate and generalizable model that can be used in clinical practice to predict time to death in cDCD donors is currently lacking. This large prospective multicenter study aims to provide a robust multimodal prediction model based on clinical, physiological, and neuroimaging parameters. In addition, it will provide valuable insights into the process of WLST in cDCD donors and its effect on timing of death (and thus donor potential). As such, this study will fill important knowledge gaps in this essential field of health care.

Accurate estimation of time to circulatory death will help clinicians and nursing staff guide grieving family members and improve the ability of medical teams to predict the chances of organ donation. This helps to manage expectations and prevent disappointment in families that are grieving but motivated to donate. In addition, it could aid in the management of organ donation procurement and transplantation team resources.

Many studies that aim to develop a new prediction tool neglect previously published models. Use of earlier data with refinement of existing models leads to more generalizable models that could be used in daily practice. In our study, we will address external validation using a large cohort with the intention to update previously published prediction tools.

Importantly, this will be the first study that extensively describes donor management in combination with end-of-life care and its impact on the timing of circulatory death in potential cDCD donors. We will provide data on the trajectory of such care during the WLST process. Valuable information on the use of sedatives and analgesics and their influence on the dying process will be obtained. Apart from the timing of death, we will also be able to analyze whether differences in end-of-life care affect family consent rates. We will demonstrate the extent of the variability in cDCD donor care.

## Appendix

Multimedia Appendix 1 Overview of demographic, clinical, and neurological parameters to be collected from potential cDCD donors. cDCD: controlled donation after circulatory death.

Multimedia Appendix 2 Overview of ventilatory, hemodynamic, and pharmacological parameters to be collected from potential cDCD donors. cDCD: controlled donation after circulatory death.

Multimedia Appendix 3 Axis showing the corresponding parameters to be collected per time point.

Multimedia Appendix 4 Peer reviewer report from the Dutch Transplant Foundation.

## Abbreviations

AUC: area under the curve
cDCD: controlled donation after circulatory death
CT: computed tomography
eCRF: electronic case report form
ICD-10: International Statistical Classification of Diseases and Related Health Problems by the World Health Organization (WHO), tenth revision
ICU: intensive care unit
LASSO: least absolute shrinkage and selection operator
STROBE: STrengthening the Reporting of OBservational studies in Epidemiology
TRIPOD: Transparent Reporting of a multivariable prediction model for Individual Prognosis Or Diagnosis
WLST: withdrawal of life-sustaining treatment
