# Supplementary material for: Prediction Model for Timing of Death in Potential Donors After Circulatory Death (DCD III): Protocol for a Multicenter Prospective Observational Cohort Study
Source: JMIR Res Protoc. 2020 Jun 23;9(6):e16733. doi: 10.2196/16733 (PMC7380979; doi:10.2196/16733)
Supplement: Multimedia Appendix 1 [file resprot_v9i6e16733_app1.pdf]

Multimedia Appendix 1. Overview of demographic, clinical and neurological parameters to be collected from potential cDCD donors.

Parameter type	Parameters included
**Demographic characteristics**	Age<br>Gender<br>APACHE II<br>APACHE IV<br>SAPS II<br>Length<br>Weight<br>BMI<br>OSAS
**Diagnosis on admission**	Traumatic brain injury<br>• Fracture of skull and facial bones<br>• Traumatic cerebral oedema<br>• Diffuse brain injury<br>• Focal brain injury<br>• Extra dural haemorrhage<br>• Intracranial haemorrhage with prolonged coma<br>• Other intracranial injuries<br>• Intracranial injury unspecified<br>• Traumatic subarachnoid haemorrhage
	Ischemic CVA<br>• Cerebral infarction<br>• Stroke not specified as stroke or infarction<br>• Occlusion and stenosis of pre cerebral arteries<br>• Occlusion and stenosis of cerebral arteries
	Post anoxic after CPR
	Subarachnoid haemorrhage<br>• ACOM<br>• PCOM<br>• PICA<br>• MCA<br>• Basilar tip<br>• Other
	Intracranial haemorrhage<br>• Parenchymal<br>• Posterior fossa cerebellar<br>• Thalamic and basal ganglia<br>• Brainstem<br>• Other

	Neoplasm of the brain<br>• Benign<br>• Malignant
	Cerebral infection<br>• Meningitis<br>• Encephalitis
	Other clinical diagnosis
**Secondary neurological injuries**[1]	Vasospasm<br>Rebleed<br>Cerebral infarction<br>Cerebral oedema<br>Epilepsy<br>Hydrocephalus<br>Cardiomyopathy<br>Compression of the brain<br>CPR
**Neurologic examination**	GCS<br>• Eye<br>• Motor (left/right)<br>• Verbal<br>Pupillary reflex (left/right)<br>Corneal reflex<br>Cough reflex<br>Oculovestibular reflex

ACOM: anterior communicating artery; APACHE: acute physiology age chronic health evaluation; BMI: body mass index; CPR: cardiopulmonary resuscitation; CVA: cerebrovascular accident; GCS: Glasgow Coma Scale; ICU: intensive care unit; MCA: middle cerebral artery; OSAS: obstructive sleep apnoea syndrome; PICA: posterior inferior cerebellar artery; PCOM: posterior communicating artery; SAPS: simplified acute physiology score II.

[1]Secondary neurological injuries are defined as damage occurring after the initial or primary neurologic injury, resulting in further clinical deterioration.
