# Supplementary material for: Prediction Model for Timing of Death in Potential Donors After Circulatory Death (DCD III): Protocol for a Multicenter Prospective Observational Cohort Study
Source: JMIR Res Protoc. 2020 Jun 23;9(6):e16733. doi: 10.2196/16733 (PMC7380979; doi:10.2196/16733)
Supplement: Multimedia Appendix 2 [file resprot_v9i6e16733_app2.pdf]

Multimedia Appendix 2. Overview of ventilatory, hemodynamic and pharmacological parameters to be collected from potential cDCD donors.

Parameter type	Parameters included
**Oxygenation and mechanical ventilation settings**	Mode of ventilation<br>• Controlled<br>• Support<br>Patient triggering the ventilator (yes/no)<br>FiO_2<br>PEEP (cmH_20)<br>paO_2 (kPa)<br>pPeak (cmH_20)<br>pMean (cmH_20)<br>SBI[1]<br>OI[2]
**Hemodynamic characteristics**	Heart rate (beats/minute)<br>MAP (mmHg)<br>CVP (mmHg)<br>CI ($l/min/m^2$)<br>LVEF (>54%, 45-54%, 30-44%, <30%)<br>Temperature (°C)
**Devices**	Pacemaker<br>ICD<br>IABP<br>Ventricular assist device<br>EVD<br>ELD
**Vasopressors and inotropes** (microgram/kilogram/minute)	Norepinephrine<br>Epinephrine<br>Phenylephrine<br>Dobutamine<br>Dopamine<br>Enoximone<br>Milrinone<br>Other
**Chronotropes and antihypertensives** (milligram/hour)	Atropine<br>Amiodarone<br>Isoprenaline<br>Ketanserine<br>Labetalol<br>Metoprolol<br>Nicardipine<br>Nitroglycerine<br>Other
**Sedation and bolus dose** (microgram/hour or milligram/hour)	Midazolam<br>Propofol<br>Dexmedetomidine<br>Clonidine

**Analgesics** (microgram/hour or milligram/hour)	Morphine<br>Fentanyl<br>Remifentanil<br>Sufentanil<br>Piritramide<br>Other
**Other medication** (milligram)	Antiepileptic<br>Scopolamine<br>Corticosteroids<br>Muscle relaxants

°C: Celsius; CI: cardiac index; cmH_20: centimetres water; CVP: central venous pressure; ELD: external lumbar drain; EVD: external ventricular drain; FiO_2: fraction of inspired oxygen; IABP: intra-aortic balloon pump; ICD: implantable cardioverter defibrillator; kPa: kilopascal; LVEF: left ventricular ejection fraction; MAP: mean arterial pressure; paO_2: partial pressure of oxygen in arterial blood; pMean: mean inspiratory pressure; pPeak: peak inspiratory pressure; OI: oxygenation index; PEEP: positive end-expiratory pressure; SBI: shallow breathing index.

[1] Shallow breathing index (SBI) is calculated by dividing the respiratory rate per minute with the tidal volume in litres such as shown after the ventilator is set on 5 cmH_2O op positive end- expiratory pressure (PEEP), 0 cmH_2O pressure support (PS) and 0.3 fraction of inspired oxygen (FiO_2) or the lowest possible settings, after 2 minutes. An SBI >105 indicates a high ventilator dependency.

[2] The oxygenation index (OI) is calculated as mean airway pressure x FiO_2 x $100/paO_2$ (kPa); mean airway pressure= peak inspiratory pressure + positive end-expiratory pressure/2.
