# Supplementary material for: Prediction Model for Timing of Death in Potential Donors After Circulatory Death (DCD III): Protocol for a Multicenter Prospective Observational Cohort Study
Source: JMIR Res Protoc. 2020 Jun 23;9(6):e16733. doi: 10.2196/16733 (PMC7380979; doi:10.2196/16733)

Multimedia Appendix 3. Axis showing the corresponding parameters to be collected per time point.

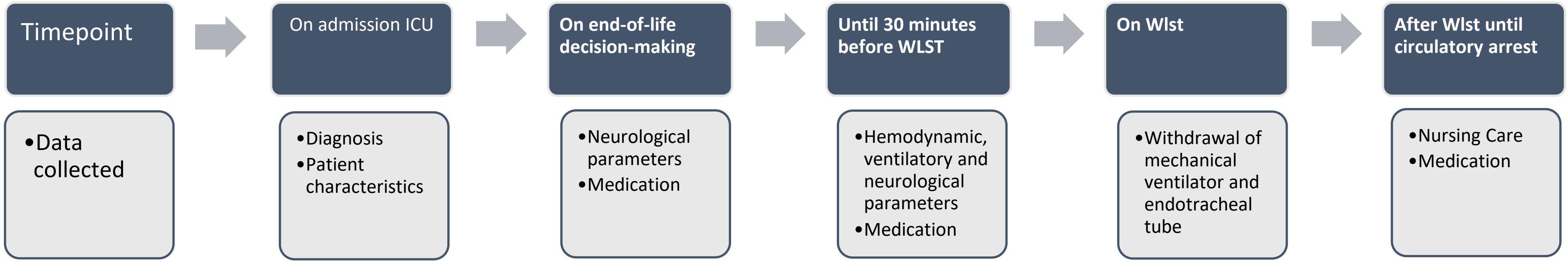

Supplement: Multimedia Appendix 3 [file resprot_v9i6e16733_app3.pdf]
