# Supplementary material for: Prediction Model for Timing of Death in Potential Donors After Circulatory Death (DCD III): Protocol for a Multicenter Prospective Observational Cohort Study
Source: JMIR Res Protoc. 2020 Jun 23;9(6):e16733. doi: 10.2196/16733 (PMC7380979; doi:10.2196/16733)
Supplement: Multimedia Appendix 4 [file resprot_v9i6e16733_app4.pdf]

NEDERLANDSE TRANSPLANTATIE STICHTING

St. Elisabeth Ziekenhuis
Drs. A. Kotsopoulos
Internist-intensivist
PO Box 90151
5000 LC TILBURG

Our reference: 21162_bhj

Leiden, 3 July 2014

Subject: First acceptance of the research proposal

Dear Ms Kotsopoulos,

Thank you for submitting the research proposal in response to the research call from the Dutch Transplant Foundation (NTS).

We have received a number of very interesting proposals. These have been discussed within the NTS Task Force. Advice was also requested from the chairman of the Dutch Transplant Society.

Your application has been selected. We do have a few additional questions.

In your research proposal you need comprehensive and complex statistics to analyse data. The assessment committee would like to see a more detailed plan how these statistical challenges will be solved; for example which departments will provide statistical support.

Many variables need to be collected from each donor, spread over five hospitals. Who will coordinate and collect the data and is there commitment from the IC departments of the participating hospitals?

Another additional question concerns the role of the NTS in this proposal. The primary intention with our call is that a proposed study will be carried out using the available NTS databases and in collaboration with some researchers of the NTS (whereby the candidate researcher would also be frequently present at the NTS office). We cannot find this information in your application. An adjustment of the proposal in this direction would be greatly appreciated.

Once we have received a response, we will provide a further opinion on your application.

With kind regards,

Drs B.J.J.M. Haase-Kromwijk,
Director NTS

Prof. dr. A.J. Hoitsma,
Medical advisor NTS

NEDERLANDSE TRANSPLANTATIE STICHTING
